# An Evaluation of the National Malaria Surveillance System of Bhutan, 2006–2012 as It Approaches the Goal of Malaria Elimination

**DOI:** 10.3389/fpubh.2016.00167

**Published:** 2016-08-19

**Authors:** Nicole West, Sonam Gyeltshen, Singye Dukpa, Kaveh Khoshnood, Sonam Tashi, Amanda Durante, Sunil Parikh

**Affiliations:** ^1^Yale School of Public Health and Medicine, New Haven, CT, USA; ^2^Vector-Borne Disease Control Programme, Department of Public Health, Ministry of Health, Gelephu, Bhutan; ^3^City of New Haven Department of Health, New Haven, CT, USA

**Keywords:** Bhutan, elimination, epidemiology, malaria, surveillance

## Abstract

**Introduction:**

Bhutan is progressing toward malaria elimination. The purpose of this evaluation was to assess the ability of the surveillance system from 2006 to 2012 to meet the objectives of the Bhutan Vector-borne Disease Control Program (VDCP) and to highlight priorities requiring attention as the nation transitions to elimination.

**Methods:**

The evaluation was conducted using the Center for Disease Control guidelines for evaluating public health surveillance systems. Data sources included a search of publically available literature, VDCP program data, and interviews with malaria surveillance personnel. Blood slide quality assurance and control through formal assessment of slide preparation and measures of between-reader correlation were performed.

**Results:**

Total malaria cases declined from 2006 to 2012. The average slide positivity rate decreased from 3.4% in 2006 to 0.2% in 2012. The proportion of non-residents in all cases increased to its highest value of 22.6% in 2012, and significant clustering in the border regions of India was noted, with Sarpang accounting for more cases than any other district from 2009 onward. Case detection was almost exclusively passive, but flexibility and sensitivity was demonstrated by the later addition of active case detection and specification of imported and locally acquired cases. Spatial data were limited to the village level, not allowing identification of transmission hotspots. For blood smears, statistical measures of between-reader agreement and predictive value were not computed. Blood smear quality was suboptimal by at least one criterion in over half of evaluated smears. Timeliness in reporting of cases was on a weekly to monthly basis, and did not meet the WHO goal of immediate notification.

**Conclusion:**

As of 2012, the national malaria surveillance system demonstrated flexibility, representativeness, simplicity, and stability. The full potential for data analysis was not yet realized. Attaining the goal of malaria elimination will require system function enhancement through increased and more accurate case detection and rapid investigation, improved health worker training and accountability, focally targeted response measures, and, in particular, the challenge of finding re-introductions of infections from India. Many such measure have been undertaken or planned as part of the next phase of the Bhutan’s National Strategic Plan.

## Introduction

Greatly intensified efforts to control and prevent malaria on a global scale have resulted in substantial reductions in malaria-associated morbidity and mortality, with global malaria deaths decreasing by 47%, and incidence by 30% from 2000 to 2013, according to World Health Organization (WHO) estimates (1, 2). Aggressive control efforts have permitted many countries to begin the transition to elimination activities (1, 3), which specifically seek to interrupt local transmission. With increasingly finer focus, programs concentrate their attentions from the national scale to the residual foci of active transmission, and finally to individual cases (4, 5).

The WHO has suggested potential epidemiologic milestones for these transitions (3, 6). The move from a control program to a pre-elimination program involves the demonstration of a blood slide positivity rate (SPR) of less than 5% for suspected malaria cases. Subsequent transition to the elimination phase requires reaching an annual parasite index (API) of less than one case per thousand population at risk per year (4). Finally, WHO certification of malaria elimination requires a country to prove the absence of any locally acquired infections for a minimum of three consecutive years (4, 6). Once this is achieved, continued vigilance is required to prevent malaria resurgence (4, 7).

The small Himalayan nation of Bhutan (population 720,629 in 2012) has had malaria control programs in place since 1964 (8). Success of these programs has been measurable, as evidenced by dramatic decreases in malaria cases from nearly 40,000 cases in 1994 to 45 cases in 2013 (9). Distribution of malaria transmission in Bhutan has been limited by its topography and climate (10). Four northern districts experience essentially no malaria transmission as a result of their elevation, and any locally identified malaria cases are likely imported from outside districts or neighboring countries. Nine districts experience seasonal transmission during the summer months, while perennial transmission occurs in the seven southern districts bordering India (11). In 2011–2013, retrospective case mapping using Geographic Information System (GIS) was carried out, and districts have been reclassified as moderate, low, potential, and no transmission zones (8). Nationally, most cases occur in August and September, following the rainy season, with a secondary peak in April.

Two-thirds of all cases occur among males, and farmers are definitively the occupational group most at risk, followed by students and laborers (8). Bhutan’s proximity to India remains a major challenge to control as porous borders, and a large number of foreign workers are constant sources for new infections. Bhutan is surrounded by states with some of the highest SPRs for *P. falciparum* in India, and many of the foreign laborers come from the highly prevalent state of Orissa (1, 12).

The *Plasmodium* species endemic to Bhutan are *P. falciparum* and *P. vivax* (11, 13). Hypnozoites of *P. vivax* represent a major challenge to elimination efforts, functioning as an asymptomatic reservoir of infection, which require additional specific therapies, namely primaquine, to eradicate these forms (14). Primaquine also targets the gametocyte stages of *P. falciparum*, making it a critical tool for reducing transmission, but its use is challenged by hemolytic anemia occurring in individuals with glucose-6-phosphate dehydrogenase (G6PD) enzyme deficiency (14, 15).

All vector-borne disease-related activities in Bhutan – including surveillance, prevention, and outbreak control – are consolidated under the Vector-borne Disease Control Program (VDCP), a division of the Department of Public Health, Ministry of Health of Bhutan. Since surveillance was initiated in 1965, reported cases reached a peak in 1994 of 39,852 malaria cases (13). Since then, the VDCP launched an aggressive malaria control strategy, strengthening and expanding earlier efforts. Following those efforts, malaria incidence in Bhutan has drastically reduced. Malaria dropped from the 16th leading cause of years of life lost in 1990 to the 46th in 2010 (16). Bhutan is in the elimination phase and is progressing well over the coming years to be certified by the WHO as malaria free (2).

Elimination rests on a country’s ability to interrupt transmission of malaria within its borders. Surveillance is lauded as a keystone for malaria elimination, and it must be tailored to the specific epidemiology, public health capacity, and disease control strategy of a given country. In the case of Bhutan, the clinical protocols and vector control activities of the VDCP have been reported previously (11, 13). The Centers for Disease Control and Prevention (CDC) has published guidelines on how to evaluate public health surveillance systems and includes assessments of data quality, timeliness in reporting, system simplicity, and data quality, among other measures (17). Using this framework, we sought to evaluate the Bhutanese surveillance system as it strives to meet the objectives of the VDCP’s 2012–2016 malaria strategic plan: (1) to reduce the number of malaria-related deaths to 0 by 2016 (shifted to 2018 in the 2015–2012 National Strategic Plan), (2) to achieve 0 local malaria transmission by 2016, and (3) to obtain WHO malaria-free certification by 2020 (17, 18).

## Materials and Methods

An evaluation of the Bhutan’s national malaria surveillance system from 2006 to 2012 was conducted using the CDC guidelines for evaluating public health surveillance systems (17). The guidelines focus on demonstrating that a surveillance system provides information that enhances public health decisions and describing system usefulness and attributes, including simplicity, flexibility, data quality, sensitivity, representativeness, timeliness, and stability. This report is structured based on these guidelines. Data were collected and analyzed in 2012 and 2013.

National malaria surveillance data from 2006 to 2012 were chosen for evaluation because these years align with the transition to the use of LLITNs and electronic record-keeping on the part of the VDCP and to achieve a finer focus on the pre-elimination years. Data were analyzed to determine numbers of blood slides collected and numbers of malaria cases by *Plasmodium sp*. and by district for each year during the study period. The evaluation included a search of PubMed, Google, and Google Scholar to identify studies pertaining to malaria in Bhutan. VDCP program data including surveillance reports, standard operating procedures, national documents were reviewed, and interviews with health workers, malaria technicians, district health officials, and VDCP personnel were conducted.

Finally, blood slide cross-check data for 2011 were analyzed including calculating the proportions meeting standards of preparation and percent agreement based on the results of the initial health center slide reading and the results from the VDCP cross-check. All data were analyzed using SAS 9.3 (SAS Institute Inc., NC, USA).

## Results

### Description of the System

As of 2012, malaria surveillance in Bhutan was a vertical system coordinated by the VDCP headquartered in Gelephu. Passive malaria surveillance is conducted year-round across the entire population of Bhutan. While little to no active case detection was conducted over this time frame, active case detection at a few hydro-power project sites had begun in 2011 (8). All health care in the country was provided by the government, so any individual seeking formal care is theoretically covered by the national surveillance system through the basic health units (BHU) or district/regional/national referral hospitals. No private health-care clinics existed as of 2012. All cases in this system are reported to the VDCP in Gelephu. Febrile patients reporting to any health center were screened for malaria, and diagnosis were made by Giemsa-stained blood slide examination for the presence of parasites and speciation according to WHO guidelines (19). Rapid diagnostic tests (RDTs) had not been widely utilized as of 2012, and microscopy confirmation is always conducted on RDT results, unless microscopy is not available. A case was defined as anyone with *Plasmodium spp*. parasites on microscopy, regardless of parasite density.

From 2006 to 2012, the approach to treatment of patients infected with *P. falciparum* required infected patients to stay in the treating health-care facility during the 3-day treatment course of artemether–lumefantrine, a first-line artemisinin-based combination therapy (ACT). Starting in 2011, a single dose of primaquine was added immediately before discharge on day 3 as a part of pro-elimination efforts. In contrast, *P. vivax* cases were treated with a combination of chloroquine and primaquine (20).

For vector control, long-lasting insecticide-treated nets (LLINs) were rolled out in 2006 to replace the earlier insecticide-treated bed nets that required regular re-impregnation. In 2010, the VDCP had achieved 96.9% coverage of households specifically targeted for LLINs, but in the endemic regions of southern Bhutan, only 77.2% of the population at risk were protected by LLINs (21). This country-wide coverage has remained stable according to indicator surveys in 2013, but achieve up to 99% in the Sarpang and Samdrup Jongkar districts (8, 22). Environmental larviciding had previously been a component of vector control strategies, but was discontinued in 2010. Focal indoor residual spraying (IRS) was conducted twice per year in areas of perennial transmission districts with a recent history of cases (23). Three sentinel sites for IRS and LLIN efficacy in the district of Sarpang had yet to find any evidence of insecticide resistance in Anopheline mosquitoes by 2012 (18).

Data collected through the national malaria surveillance system were analyzed by the VDCP and used to inform the planning of CDC activities at the local level, including targeted IRS and heightened efforts of community outreach, environmental management, and vector studies. Quarterly reports were sent from the VDCP to health centers, and a formal annual report was prepared for a review of the previous year with Ministry of Health officials. The village-level criteria used by the VDCP to determine where to conduct the twice-yearly (March and September) focal IRS were as follows: (1) *P. falciparum* cases accounting for over 30% of total malaria cases during the three preceding years, (2) API above 10 cases per 1,000 population, (3) SPR above 10% for preceding 3 years, and (4) deaths due to malaria (23, 24). The 2012 guidelines included the addition of focal IRS following a malaria index case if that area did not qualify for IRS under the above criteria (18).

### Malaria Surveillance Results from 2006 to 2012

**Figure 1:**
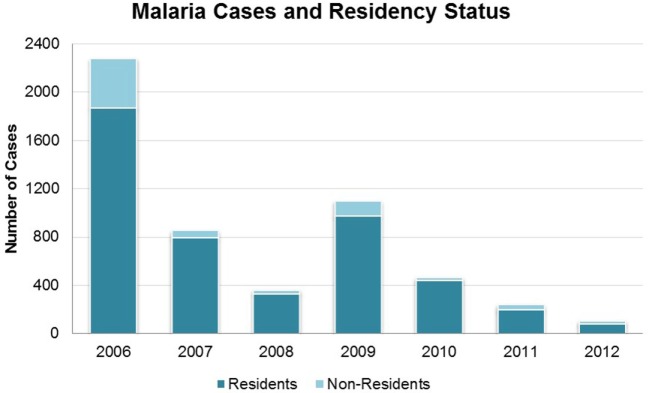
**Malaria cases in Bhutan 2006–2012 by residency status, as defined during this time period**. All cases are microscopy-confirmed.

Overall, total malaria cases (*including cases among both residents and non-residents of Bhutan*) declined from 2,276 in 2006 to 106 in 2012 (Figure 1). The exception was 2009 when there was a single-year increase to 1,098 cases; in 2010 cases dropped again to 465. Health workers and VDCP officials suspected this was due to waning efficacy of LLINs as the increase was not localized, but was rather seen across the country. Additional LLINs were distributed in early 2010. The district of Sarpang (in the region of perennial transmission) consistently had high numbers of cases, and accounted for the majority of cases from 2009 onward. Over the 7-year period, 15 malaria-attributable deaths occurred, decreasing from four in 2006 to a single death in 2012. No deaths were reported in 2013, suggesting that the VDCP’s zero-mortality goal is achievable (8). Further reductions in malaria cases were reported in 2013, with 45 microscopy-confirmed cases (8).

**Figure 2:**
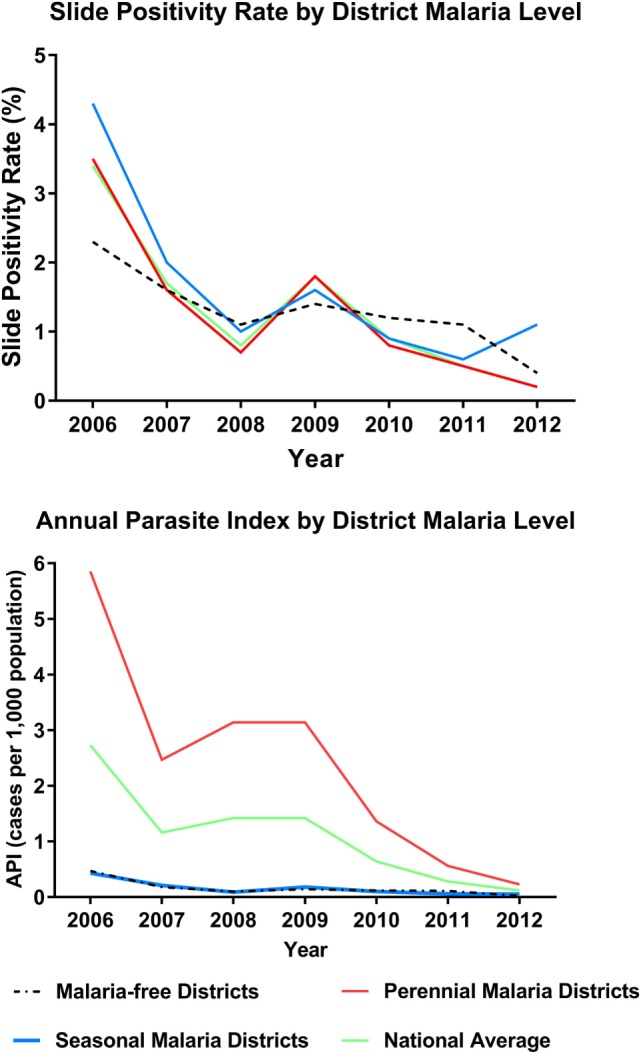
**Indicators of program progress: slide positivity rate (SPR) and annual parasite index (API) from 2006 to 2012**. Includes cases in residents and non-residents. Figures further stratified by district level of transmission intensity (no transmission, seasonal, perennial).

Slide positivity rate and API both decreased over this period, surpassing WHO milestones for program transition to pre-elimination and elimination [Table 1; Figure 2 (4)]. The SPR (*including cases among both residents and non-residents of Bhutan*) was below 5% for all three levels of regional transmission for the duration of the 7-year period, with the national average decreasing from 3.4% in 2006 to 0.2% in 2012. API fell below one case per thousand population in districts with perennial transmission in 2011, while API for regions of seasonal and no malaria transmission were less than 0.5 throughout.

**Table 1:** **Total numbers of blood slides examined and slide positive rate, by year**.

	Year						
	2006	2007	2008	2009	2010	2011	2012
Blood slides examined	66,079	51,446	47,268	62,342	54,616	44,481	42,512
Slide positivity rate	3.4%	1.7%	0.8%	1.8%	0.9%	0.5%	0.2%

Importantly, designation of resident/non-resident status from 2006 to 2012 did not take into account travel history or other case investigation history. Thus, this designation did not equate to local versus imported case definitions. In late 2012, case definitions were adjusted to consider the likely source of malaria acquisition, an important step in reaching the goal of elimination. Using the prior definitions, non-residents comprised between 6.2 (in 2010) and 22.6% (in 2012) of all cases (Figure 1). However, these cases were not evenly distributed across all districts; rather, there were more transient, localized effects in particular districts, changing from year to year. In 2011, every non-resident case was detected in Sarpang, where they accounted for 45 of 148 total cases (30.4%). In 2012, cases among non-residents were only reported in Wangdue and Sarpang, accounting for 7 of 15 (46.7%) of cases in Wangdue and 17 of 67 (25.4%) of cases in Sarpang. Data from 2013, suggest a shift to more cases occurring in non-residents than in Bhutanese, on a national scale (8).

**Figure 3:**
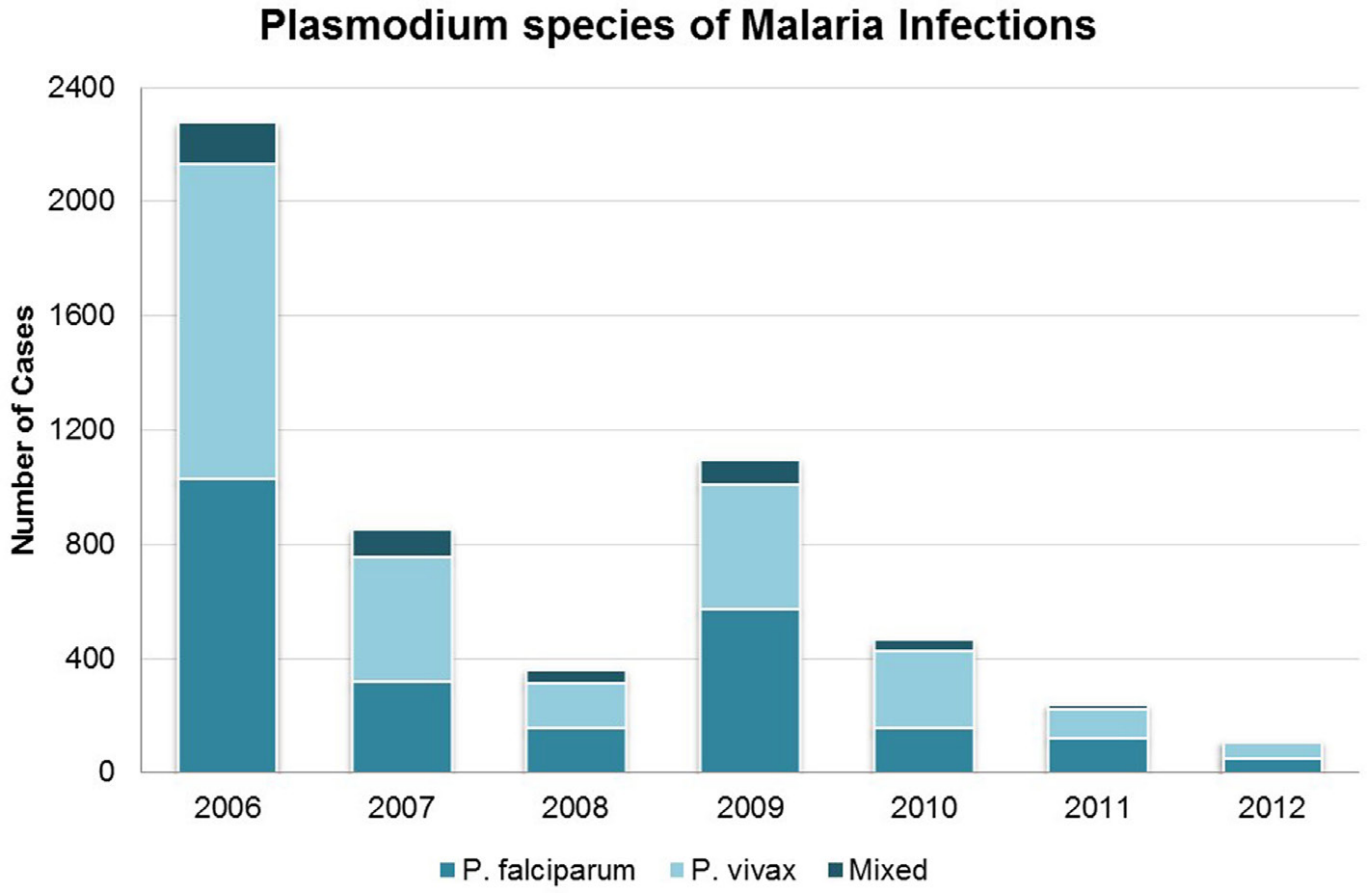
**Malaria cases in Bhutan 2006–2012 by species of *Plasmodium***. Categories are *P. falciparum, P. vivax*, and mixed species infection.

**Figure 4:**
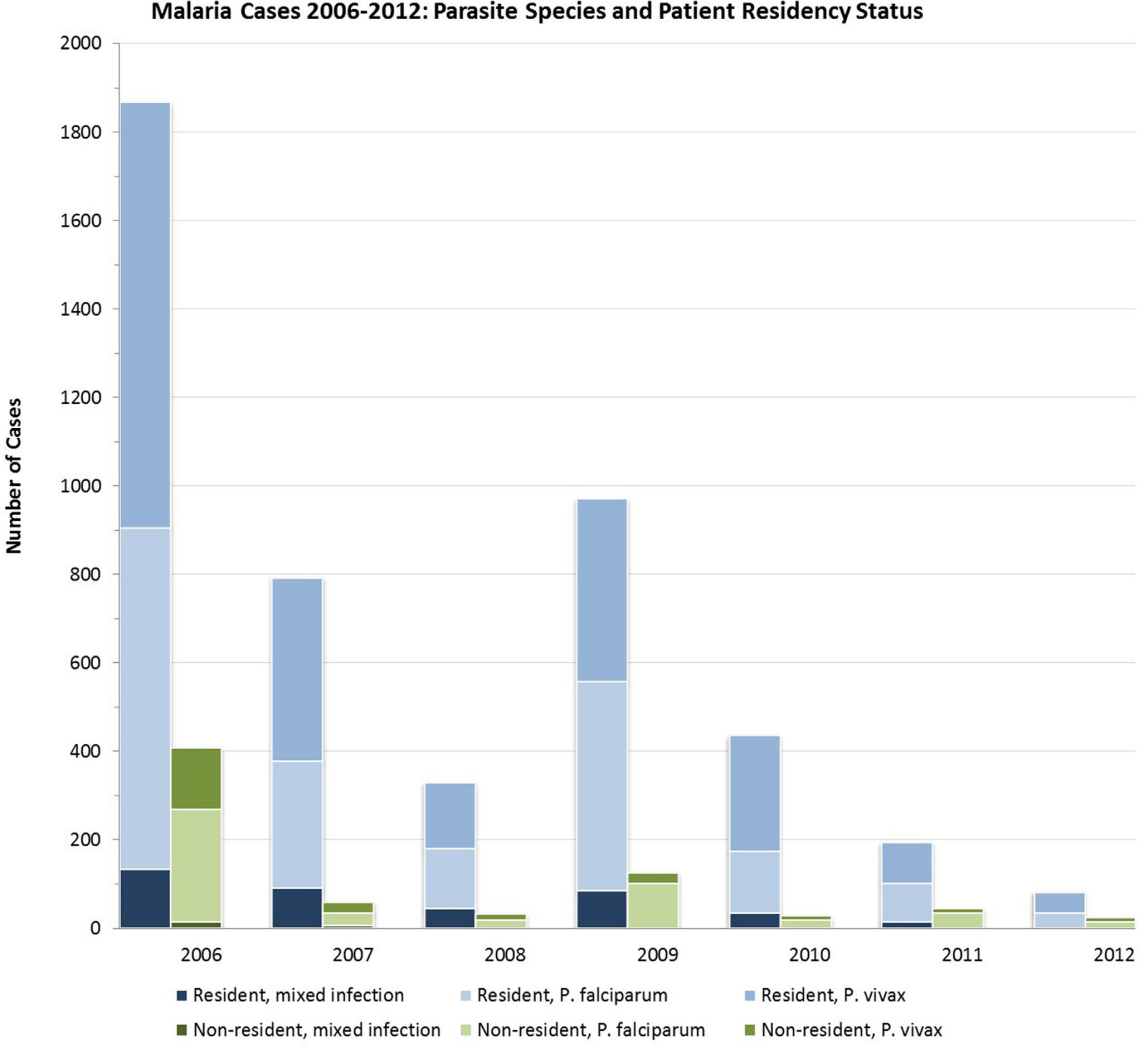
**Malaria cases in Bhutan 2006–2012 by species of infection among residents and non-residents**.

Proportions of infections by species (*P. falciparum, P. vivax*, or mixed infection) did not vary significantly over time (Figure 3). From 2006 to 2012, *P. vivax* accounted for nearly two-thirds of all infections in seasonal transmission and malaria-free districts, while an additional 22.3–25.0% were due to *P. falciparum* and 12.3–13.0% were mixed. In the perennial transmission districts, infections were more evenly split between *P. falciparum* and *P. vivax* (43.1 and 48.6%, respectively; 8.3% mixed), perhaps due to the high burden of *P. falciparum* in neighboring Assam (25, 26). Figure 4 shows the parasite species and patient residency status over the evaluation period.

### System Usefulness and Attributes

The system alerted VDCP officials to the 2009 malaria outbreak – leading to the distribution of additional LLINs in 2010 – and had tracked the more local trends, such as the reduction in incidence in Samdrupjonkar, which had formerly had rates comparable to those of Sarpang. Sarpang was identified as the major contributor of cases, and the district was targeted for additional control efforts. VDCP were able to utilize. Data, though useful in the scenarios above, were not analyzed to its full potential. For example, spatial data were collected only at the village level, though an even finer scale of spatial analysis would be useful for the identification of transmission hotspots (27, 28). Importantly, beginning in 2011, the VDCP had begun to introduce retrospective case mapping using GIS to identify evidence-based strategies for targeted interventions, an important advance for achieving and sustaining elimination (8).

### Data Quality and Positive Predictive Value

Health centers that had treated febrile patients within the last month were required to send a portion of blood slides, which had been collected to the VDCP for cross-checking by a trained professional. Health centers with fewer than 50 fever patients were required to send in all of their slides, while those with 50 or more were required to send all of their positive slides and 10% of their negative slides for cross-checking. Slides were reviewed by an official at the VDCP with more advanced parasitology training to assess accuracy in initial diagnosis. The official was blinded to the initial result while slides were examined for the presence of species of parasites. The size, evenness, staining, and cleanliness (dichotomously, acceptable or not), as well as whether the slide includes both thick and thin smears as required by VDCP protocols (23), were recorded.

The inclusion of the blood slide cross-checking process allowed for evaluation of the validity of diagnoses. Of 2,514 blood slides analyzed during the 2011 cross-check process, 1,160 (46.14%) were judged by the VDCP official to be of excellent quality (programmatically defined as free from any staining problems or blemishes, and containing both thin and thick smears). Over half of smears were judged to be sub-optimal in at least one characteristic. The greatest deficit was that only 56% of slides had been prepared with both a thick and thin smear (38% only had a thick smear prepared). Lower than expected numbers of blood slides sent in for confirmation may suggest a lack of acceptability in this portion of the process. Other deficits were much less frequently encountered (91% of smears were of a normal size, 88% were evenly prepared, and 93% were stained satisfactorily). Sixty slides (2.4%) arrived broken at the VDCP and could not be read for cross-checking.

At the time of the assessment, false positives and false negatives were counted, but no percentages or measures of agreement were computed. The WHO recommends calculating percent agreement between health worker and validator reading for the presence of any parasites and for the presence of *P. falciparum* specifically (29). Where slide readings from both the original health worker and the VDCP professional during cross-check could be compared, agreement of parasite presence or absence of parasites was >97% for districts of all three transmission levels (Table 2). However, in 2011, readings from two health centers accounted for 12 of 19 false positives (all of which were identified as *P. vivax* infections). Positive predictive value (PPV), or the proportion of positive results that represent true positives, was not explicitly calculated, an important measure as true incident cases continue to decline. Similarly, negative predictive value (NPV), or the proportion of negative results that represent true negatives were not calculated, an important measure as missing cases poses a risk for severe disease sequelae and onward transmission.

**Table 2:** **Agreement of slide readings during cross-check in 2011**.

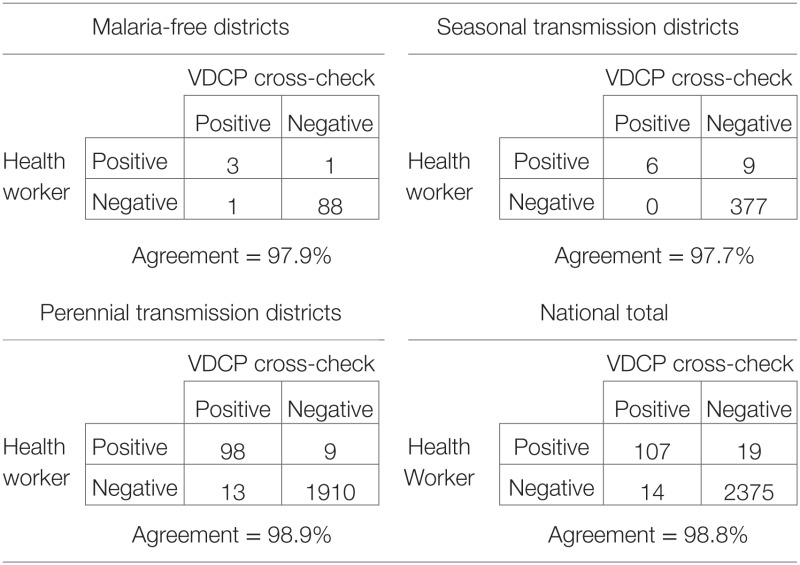

### Sensitivity and Acceptability

Two major factors determine the sensitivity of the system: patients’ health care-seeking behavior and the ability to properly assess blood slides, which was discussed earlier (30). According to the 2009 National Malaria Indicator Survey and Bed Net Knowledge, Attitude, and Practice Study (MIS & KAP) of 796 households, 745 (93.6%) knew what malaria was, 663 (83.3%) identified fever (the criteria for blood screening in health centers) as a major symptom, and 83.5% reported seeking care within 24 h of a fever. Regarding treatment, 739 (92.8%) respondents said that malaria could effectively be treated by visiting a health center, while only 3 (0.4%) respondent said treatment could be achieved by visiting a traditional healer or astrologer (21). Increasing public awareness and encouraging individuals to go to health-care centers when they experience a fever has been a focus of the VDCP’s malaria control strategy. More recent indicator surveys show progress in this area (22). The sensitivity of the system was also demonstrated with the piloting of active case detection in hydro-power project sites beginning in 2011, and the addition of both reactive and an expansion of active case detection activities is a part of Bhutan’s 2015–2020 strategy for malaria elimination (3, 8, 31).

### Timeliness and Flexibility

Currently, the WHO recommends that malaria eliminating countries should target the reporting of all cases to the district or intermediate level with a copy to the central level immediately, ideally the same day, by telephone, SMS, or e-mail (32). All health center records and surveillance reports were completed on paper and delivered by post, fax, or by car to the VDCP. Health centers sent in reports to the VDCP every month, except for those in the seven endemic districts which reported weekly by phone (23). Reports for a given month were to be received by the VDCP by the beginning of the second week of the following month, although some reports were received up to 2 months after this due to distance of particular BHU or seasonally-disrupted terrain. All data were received by the Information Unit of the VDCP, and since 2006 had been entered into Epi Info (Centers for Disease Control and Prevention, Atlanta).

The CDC defines flexibility as a system that “can adapt to changing information needs or operating conditions with little additional time, personnel, or allocated funds” (17). Flexible systems are also those that can accommodate “changes in case definitions or technology and variations in funding or reporting sources” (17). Structurally, Bhutan’s system appeared able to accommodate new components such as phone reporting and reactive case detection. As described above, Bhutan now plans to introduce more active case detection and more stringent definitions of local versus imported cases. Elimination efforts require on-going review of the progress of the program and evaluation of the performance of the surveillance system (32). To this end, the system has demonstrated flexibility through external reviews and the continued evolution of its malaria strategy over time. In addition, there is the potential for the malaria surveillance system to demonstrate its flexibility in other ways, such as the expansion to include surveillance for other diseases notifiable diseases. However, no indication of such expansion was evident at the time of this review.

### Representativeness and Simplicity

Surveillance was complete across the country and included the provision of reports even in the absence of cases identified during the period. Bhutan lacks a private health-care sector, and all Bhutanese attend government health-care centers, all of which report to the VDCP. Although structurally consistent throughout the country, the system does not appear to be completely uniform in its quality of coverage, with BHUs in the endemic districts having higher numbers of blood slides collected. However, due to the climate of Bhutan and the ecological restriction of the mosquito vector to the southern districts, imperfect representativeness may be less detrimental.

The system as it currently stands is quite simple. As a result of the VDCP’s functional autonomy, there are no intervening entities between the point of diagnosis and the center of data receipt and analysis. During the time of this assessment, a handful of private laboratories were beginning to emerge, which could impact on simplicity, although no data were obtained from these settings.

### Stability

Stability is defined as “the reliability (i.e., the ability to collect, manage, and provide data properly without failure) and availability (the ability to be operational when it is needed) of the public health surveillance system” (17). In many ways, the current simplicity and low reliance on technology render the malaria surveillance system quite structurally stable and resilient in the face of obstacles such as power outages, though the growth of internet and other mobile technology poses new challenges to stability. An additional identified challenge will be how to sustain high quality surveillance and aggressive control efforts as funding decreases. The VDCP and its malaria control activities are funded by the Royal Government of Bhutan, the Government of India, the WHO, and the Global Fund (GFATM) (31). International funding had suffered as a result of the global financial crisis over this time period (33).

## Discussion

We aimed to assess the malaria surveillance system in Bhutan from 2006 to 2012 using CDC guidelines for the evaluation of public health surveillance systems. It should be noted that since our assessment, many of the recommendations described herein have been either enacted or planned as described in Bhutan’s updated 2015 to 2020 National Strategic Plan (8). Notably, a recent external review was conducted in collaboration with the WHO, and recommendations from this review are outlined in the strategic plan. Overall, from our assessment, the malaria surveillance system of Bhutan appeared strong and produced data that was useful and of good quality. The performance of the system has been critical to the major reductions to malaria morbidity and mortality in Bhutan. However, elimination will require the system to function at an even higher level, and measures are underway to meet this challenge (Table 3).

**Table 3:** **Priority action recommendations**.

*Priority actions*
*Enhanced data management and analysis*: The surveillance system utilizes a fraction of the information, which is collected, and harnessing this information more effectively could aid in pro-elimination efforts. Addition of a calculation, such as percent agreement of slide readings, can provide a way to compare districts and the national average, to assess progress over time, and to meaningfully communicate with outside public health entities.
*Optimize diagnostic capacity*: Additional immediate training for local health workers is a necessity, as well as a more rigorous attention to individual worker performance. Routine refresher training should be required. Competency will be difficult to maintain in an era of elimination, so continued monitoring will be necessary.
*Improved timeliness*: Standardizing telephone reporting is a potential avenue. The VDCP may consider incorporating the proportion of *P. falciparum* infections with gametocytes present as an indicator of timeliness of patient diagnosis for use during the cross-check process. Timeliness will likely improve with expansion of digital access and potential SMS based reporting.
*Active case detection*: This should include active screenings in the vicinity of locally acquired infections, as well as a more pro-active approach toward imported infections. Active screenings as a prerequisite for foreign workers would be a significant step in the control of cross-border malaria.
*Formalization of cross-border collaboration*: A major obstacle for the elimination of malaria in Bhutan will be ongoing reintroductions of malaria through porous borders and migrant laborers. Unified efforts between India and Bhutan are required.
*Expansion of surveillance apparatus to include other diseases*. The successes of the malaria surveillance system can be leveraged in the control of emerging vector-borne diseases in Bhutan, such as dengue, leishmaniasis, and chikungunya.
*Additional studies into G6PD prevalence and vector ecology*: Though not detailed in this assessment, G6PD prevalence must be determined; the hazard of using primaquine as a standard treatment in the absence of this knowledge is great, and monitoring should be conducted adverse reactions. Research into the ecological epidemiology of malaria in Bhutan, including vector incrimination and environmental drivers of transmission, would allow targeted vector control as well as forward-looking risk assessment for challenges such as climate change

Our assessment of blood smear cross-check data from 2011 revealed a deficiency in diagnostic accuracy. This will require the addition of measures of reader agreement, additional training for local health workers, more rigorous attention to individual worker performance, and remedial measures when required. The predictive power of negative results is of particular significance because of consequences to an individual and as a source for future infections. The existence of a quality assurance component is a great strength of the structure of this surveillance system, but it is only of value if the results are utilized. Health worker accountability should be improved. While health centers that have repeated errors identified through the cross-check process are invited to send health workers to refresher trainings at the VDCP, workers are often not sent. This process should be formalized and made to be a requirement with set expectations for performance (29). The WHO and others recommend a system of on-going training and assessment of microscopists for competency in slide preparation and reading of both parasite presence and species, coupled with a clear protocol for remedial measures if expectations are not met (29, 34). District health centers must be encouraged to continue to send in the required number of blood slides as the blood slide cross-checking process is the primary means by which the VDCP can assess the sensitivity and specificity of diagnoses. Plans for the establishment of a National Quality Assurance system for malaria diagnosis should assist in making these adjustments (8). In addition, as new private laboratories are emerging in Bhutan, the VDCP should ensure that similar proficiency testing occurs in these centers as well.

Second, more sophisticated data management and analyses were required. The malaria surveillance system in Bhutan collected more information than was utilized, and harnessing this information more effectively would have aided in pro-elimination efforts. At a national level, training of personnel in statistics and epidemiology represented a significant challenge. As this is being addressed, data such as slide-reader agreement should be added to standard analyses. In addition, SPR calculated monthly at BHUs (instead of only at the year-end) may help to better understand the local transmission as compared to the raw case counts. These calculations can provide a way to compare districts and the national average, to assess progress over time, and to meaningfully communicate with outside entities concerned with public health.

Third, timeliness of identifying and treating cases is an area for improvement. The WHO has suggested using the proportion of *P. falciparum* infections with gametocytes present as a timeliness indicator, as treatment of *P. falciparum* infections within the first 6 days from the onset of symptoms is felt to be sufficient to prevent the development of gametocytes (6). The VDCP therefore may consider incorporating this indicator into its protocols. Ideally, the presence of gametocytes and resulting proportion should be recorded and calculated at the point of care.

In addition, to improve timeliness of reporting, significant enhancements will be needed to meet the WHO goal of “immediate” reporting of cases to national malaria programs (32). In the short-term, phone-based reporting, which is already regularly practiced with success in the southern districts, could be expanded to the seasonal transmission districts, potentially as a transient feature activated during the high case season. In the mid- to long-term, electronic reporting will enhance timeliness, but improved funding and training to expand internet access, acquire computers, and attain necessary technical expertise levels is required. With the advent of GIS case mapping, alongside the growth of mobile and internet capacity, significant improvements in timeliness is expected. Several countries have established SMS reporting systems with much success, including Sri Lanka in 2009 (35–38).

Fourth, from 2006 to 2012, the program functioned primarily in a passive detection capacity, but programmatic changes toward both reactive and active case detection are now underway (8). In several other countries, reactive case detection has successfully been used to focally increase sensitivity by requiring active blood screens of household members and other residents within a defined radius of any newly identified malaria case (35, 39). In addition, since 2012, case definitions have been modified to appropriately designate cases as imported or locally acquired, data which are critical to achieve and sustain elimination (26, 40). The addition of genetic analysis of parasites to confirm whether infections are locally acquired or imported would not seem to substantially challenge the structure of the current system (41). With such changes, the surveillance system will have to tighten its coverage, as reintroductions from porous borders and migrant workers will be an ongoing major threat to both achieving and sustaining elimination. The results of these case investigations then must be mined at the program level to attack foci of transmission and to implement targeted measures addressing populations identified as having the greatest risk, due to geographic location or occupational hazard (1, 27). Importantly, the use of spatial decision support systems, including GIS, is now underway, and will aid the in identifying hotspots and hotpops, and the deployment of targeted intervention strategies. In addition, targeted active screenings of workers at hydro-power project sites is underway. While this is an important step, care must be taken to ensure that workers do not become averse to seeking care for fear of loss of work privileges. In addition, innovative means of exposure reduction will have to be developed to prevent infection in other at-risk groups such as farmers, who are not protected by the primary vector control activities of IRS and LLIN use. In this case, the malaria surveillance system has been successful in identifying an at-risk population, but this information has not been translated into public health action.

The Roll Back Malaria initiative suggests that countries with highly malarious borders may choose to postpone elimination efforts until neighboring countries are able to control their high incidence levels (3) Delaying elimination efforts until control is achieved in India would mean a substantial delay. However, in the absence of effective surveillance and health service delivery in Assam, this state and its neighbors will continue to act as a source for re-introduction of infections in southern Bhutan (42). Cross-border collaborations have been limited, but they will have to be a priority for both countries to see case reductions in this area. Available funding for such collaborations, however, is limited (43). The Asia Pacific Malaria Elimination Network raises awareness of the collective progress of and challenges for elimination in the region acts as a catalyst to achieve consensus on important elimination issues, such as mobility and migration (9).

Finally, and more broadly, the successes of the VDCP and the malaria surveillance system can be leveraged in the control of emerging vector-borne diseases in Bhutan such as dengue, leishmaniasis, and chikungunya. Integrated vector management strategies can be used to progress toward elimination even as the spread of these other diseases is controlled, and the surveillance apparatus for malaria could be expanded to fill the present void of systematic surveillance of other vector-borne diseases.

Our evaluation had several limitations. The analysis of blood slide quality data could have been strengthened by an expert review of a random sample of all blood slides (both those sent in to the VDCP and those collected at BHUs but not sent in) as well as a direct assessment slide reading proficiency of local health workers and VDCP officials. Nevertheless, we believe that our assessment of the design and function of the surveillance system is well-founded despite these potential limitations.

Bhutan has seen major reductions in malaria morbidity and mortality since the 1994 peak of cases, and a substantial dwindling from 2006 to 2012, and major advances evident in 2013 data. Surveillance has played an important role in the ability of the VDCP to provide high quality, targeted control services to the foci of transmission during this period. Bhutan is continuing to develop and expand its surveillance system, and the addition of targeted active and reactive case detection, along with GIS data will be beneficial. The malaria elimination community must ensure that Bhutan does not become a victim of its own success, and that donors who have provided substantial support in the past do not turn their attention to other countries. Studies of malaria resurgence have found that nearly all instances of resurgence are due to the slackening of control efforts, which are themselves primarily due to insufficient resources (7, 40). In the absence of any external reduction in regional transmission potential, the activities used to achieve malaria control or elimination must be maintained (44). Despite such challenges and the potential of re-introductions of infections, elimination in Bhutan in the next few years appears possible with the continued responsiveness and adaptability of its surveillance system that has been demonstrated to date.

## Author Contributions

NW, SG, SD, ST, and KK conceived of the study. NW, SG, SD, AD, and SP obtained and analyzed the data. NW, KK, SD, and AD wrote the manuscript.

## Conflict of Interest Statement

The authors declare that the research was conducted in the absence of any commercial or financial relationships that could be construed as a potential conflict of interest.
